# MPT0G066, a novel anti-mitotic drug, induces JNK-independent mitotic arrest, JNK-mediated apoptosis, and potentiates antineoplastic effect of cisplatin in ovarian cancer

**DOI:** 10.1038/srep31664

**Published:** 2016-08-16

**Authors:** Han-Li Huang, Min-Wu Chao, Ya-Chi Li, Li-Hsun Chang, Chun-Han Chen, Mei-Chuan Chen, Chun-Chun Cheng, Jing-Ping Liou, Che-Ming Teng, Shiow-Lin Pan

**Affiliations:** 1 The Ph.D. Program for Cancer Biology and Drug Discovery, College of Medical Science and Technology, Taipei Medical University, Taipei, Taiwan; 2 Pharmacological Institute, College of Medicine, National Taiwan University, Taipei, Taiwan; 3 Department of Pharmacology, School of Medicine, College of Medicine, Taipei Medical University, Taipei, Taiwan; 4 Ph.D. Program for the Clinical Drug Discovery from Botanical Herbs, College of Pharmacy, Taipei Medical University, Taipei, Taiwan; 5 Graduate Institute of Pharmacognosy, College of Pharmacy, Taipei Medical University, Taipei, Taiwan; 6 School of Pharmacy, College of Pharmacy, Taipei Medical University, Taipei, Taiwan

## Abstract

Developing new anticancer agents against ovarian cancer is an urgent medical need. MPT0G066, a novel synthetic arylsulfonamide compound, was shown to inhibit cell growth and decrease viability in human ovarian cancer cells. MPT0G066 induced arrest of the cell cycle at the multipolyploidy (MP) phase in SKOV3 and at the G_2_/M phase in A2780 cells, while increasing the proportion of cells in the subG_1_. Additionally, MPT0G066 induced c-Jun-NH2 terminal kinase (JNK) activation, influenced cell cycle regulatory and Bcl-2 family proteins, which triggered intrinsic apoptotic pathways through cleavage of caspase-3, -7, -9, and poly-(ADP-ribose) polymerase (PARP). Flow cytometry analysis of p-glycoprotein (p-gp) function showed that MPT0G066 was not a substrate of p-gp. Additionally, it was shown that MPT0G066 could decrease cell viability in multiple-drug-resistant human ovarian cancer cells. Furthermore, the combination of MPT0G066 and cisplatin presented a synergistic cytotoxic effect against ovarian cancer cell lines *in vitro*. MPT0G066 also significantly suppressed the growth of ovarian carcinoma and potentiated the antineoplastic effects of cisplatin *in vivo*. In conclusion, these findings indicate that MPT0G066 can be a potential anticancer agent against ovarian cancer that worthy of further development.

Ovarian cancer is the second most common cause of gynecologic cancer-related death in women in the world, which with relatively high mortality rate due to the difficulty to diagnose at an early stage. About 75% of ovarian cancer patients with stage III or IV receive first-line therapy of combination of platinum and taxane. Unfortunately, most patients with advanced disease eventually experience cancer recurrence and chemotherapy resistance after six months of treatment. Additionally, alternative therapies such as gemcitabine or cyclophosphamide exhibit the problem of the risk of toxicity^1^,^2^. Therefore, the discovery of new drugs is an unmet need for patients with advanced ovarian cancer.

C-Jun-NH_2_ terminal kinases (JNKs) are members of the mitogen-activated protein kinase (MAPK) family, and are involved in the cellular stress response that determines whether the cell will survive, differentiate or undergo apoptosis. Some studies have indicated the multifaceted role of JNK activity in biological functions that acute JNK signaling promotes cell survival, but prolonged JNK activation is associated with apoptosis^3^ and the increase in JNK-affected c-Jun activity was found to promote tumorigenesis in some types of cancer. Inhibition of JNK activity reduced the growth of cancer cells in small-cell lung cancer and breast cancer cells; however, cytotoxic drug-induced JNK activation was found to decrease cancer cell proliferation leading to cell apoptotis^4^. Also, previous research has indicated that microtubule-interfering agents activate JNK signaling to downregulate Bcl-2 and promote apoptosis^5^,^6^,^7^.

Dysregulation of the cell cycle cause cancer cells to sustain proliferative signaling and evade growth suppressors, thereby enabling replicative immortality. Anti-mitotic agents, including microtubule-targeting agents and mitosis kinase inhibitors, are a class of drugs that target cell cycle process. Treatment with anti-mitotic agents directly influences cell division through disruption of microtubule dynamic, mitosis programming factors (cyclin B1 complex) or the activation of the mitotic spindle checkpoint (Aurora kinases and PLK-1)^8^,^9^. Histone H3 phosphorylation is tightly correlated with chromosome condensation during both mitosis and meiosis and is considered as a mitotic marker. Subsequently, these stressors signal the triggering of the apoptotic pathway through dysregulation of the Bcl-2 protein family^9^. However, several studies indicate that cancer cells acquired resistance to anti-mitotic agents (*e.g.* paclitaxel and cisplatin) through overexpression of p-glycoprotein and glutathione^10^. Thus, investigate new therapeutic agent to overcome drug resistance is also an important issue at an early stage of drug development.

This research focused on the therapeutic potential of MPT0G066, a novel synthetic arylsulfonamide compound, against ovarian cancer. MPT0G066 showed anti-proliferation, cytotoxic activity and induced JNK-mediated mitotic arrest in ovarian cancer cell lines SKOV3 and A2780 *in vitro.* In addition, we demonstrated that MPT0G066 could exhibit a cytotoxic effect in multi-drug resistant ovarian cancer cells and evaded the chemoresistance by being a non-p-gp substrate. Furthermore, MPT0G066 potentiated the antineoplastic effect of cisplatin *in vitro* and *in vivo*. Based on our finding, we suggest that MPT0G066 might be a potential therapeutic agent against human ovarian cancer.

## Results

### MPT0G066 shows potent antitumor activity and causes cell cycle arrest in ovarian cancer cells

**Figure 1:**
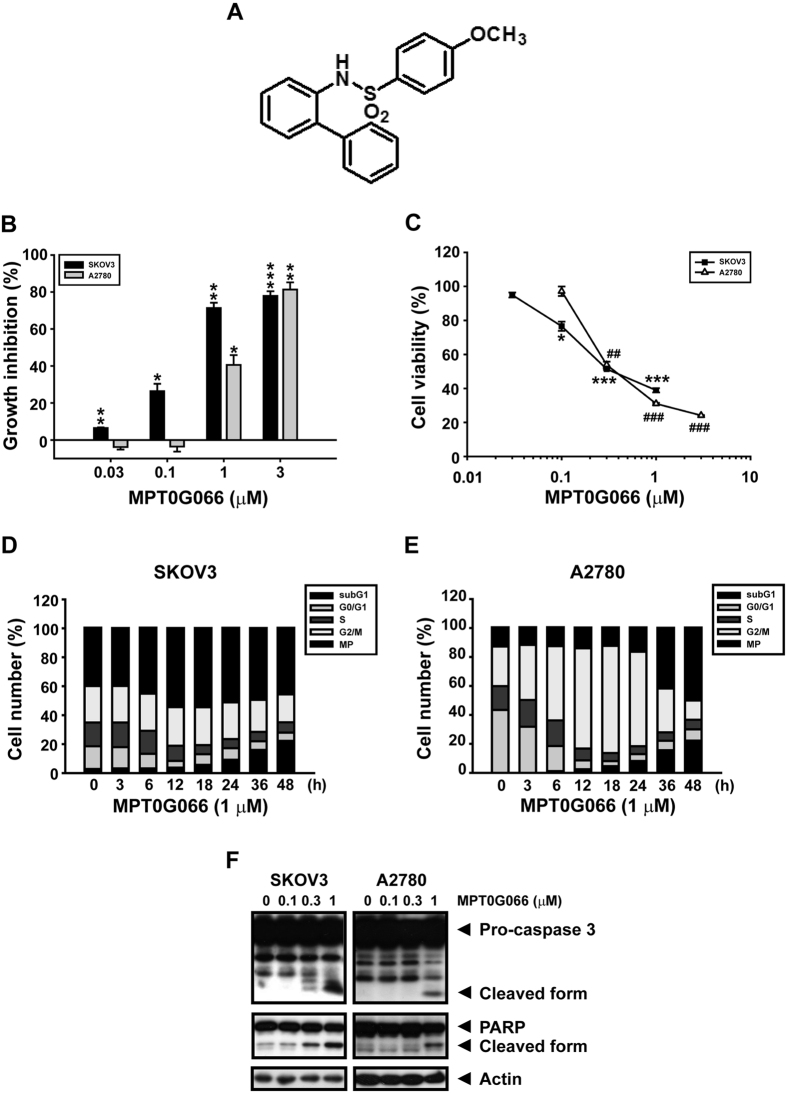
MPT0G066 reveals a potent antitumor activity in ovarian cancer cells. (**A**) The illustration represents the chemical formula of MPT0G066. (**B**) Cell proliferation was determined by sulforhodamine B (SRB) assay. (**C**) Viable cells were analyzed by MTT assay. (**B**,**C**) SKOV3 or A2780 cells were treated with the indicated concentrations of MPT0G066 (0.01–10 μM) for 48 h. (**D**) SKOV3 or (**E**) A2780 cells were treated with 1 μM MPT0G066 for different time intervals. Cell cycle was analyzed by flow cytometry after propidium iodide staining. (**F**) SKOV3 or A2780 cells were treated with the indicated concentrations (0–1 μM) of MPT0G066 for 48 h. The whole cell lysates of SKOV3 or A2780 were subject to western blot analysis with caspase-3, PARP and actin. *P < 0.05, ** or ^##^*P* < 0.01 and *** or ^###^*P* < 0.001 compared with vehicle-treated cells.

First, we determined the antitumor activity of MPT0G066 in two ovarian cancer cell lines, SKOV3 and A2780. Using SRB assay, it was shown that MPT0G066 inhibited ovarian cancer cell proliferation with a GI_50_ value of 0.21 μM for SKOV3, and a GI_50_ value of 0.41 μM for A2780 (Fig. 1B). Compared with our previous finding, MPT0G066 exert its antiproliferative effect most efficaciously in ovarian cancer cells^11^. Further, MTT assay showed that MPT0G066 repressed SKOV3 and A2780 cell viability with an IC_50_ value of 0.44 μM and 0.45 μM respectively (Fig. 1C). In addition, MPT0G066 decreased the cell viability with an IC_50_ value of 6.16 μM in human umbilical vein endothelial cell (HUVEC) (Supplementary Fig. S1), which represented the safety of MPT0G066 against normal cell under anti-tumor effective dose in ovarian cancer cells.

Next, we investigated the effect of MPT0G066 on the cell cycle using flow cytometry analysis. Both cell lines were treated with 1 μM MPT0G066 for different time intervals. In SKOV3 cells with p53 mutation, the population of the multipolyploidy (MP) phase increased at 12–24 h (Fig. 1D). On the contrary, MPT0G066 promoted mitotic arrest in A2780 cells, which harbor wild-type p53, at 12–24 h (Fig. 1E). Finally, MPT0G066 increased the population of subG1 cells and triggered a caspase-dependent apoptosis pathway in both cell lines at 36–48 h (Fig. 1D–F). As a result, MPT0G066 induced mitotic arrest, and subsequently apoptosis, in ovarian cancer cells.

### MPT0G066 activates phosphorylation of JNK and regulates both mitotic proteins and the Bcl-2 protein family

**Figure 2:**
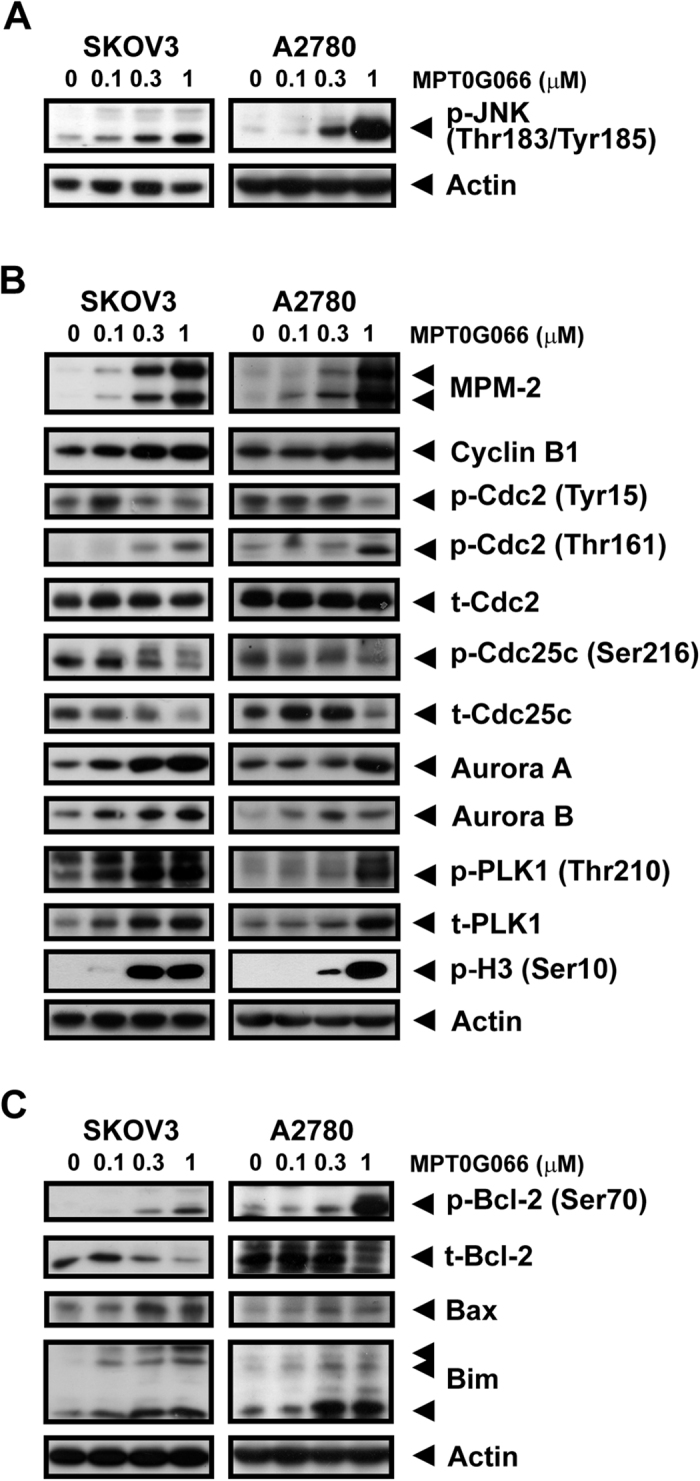
MPT0G066 increases phosphorylation of JNK and alters the regulatory proteins of G2/M transition and Bcl-2 protein family. SKOV3 or A2780 cells were treated with the various concentrations of MPT0G066 (0–1 μM) for 18 h. The whole cell lysates of SKOV3 or A2780 were subject to western blot analysis with (**A**) phosphorylated JNK (Thr-183 and Tyr-185), (**B**) MPM-2, cyclin B1, total or phosphorylated cdc2 (Tyr-15 and Thr-161), total or phosphorylated cdc25c (Ser-216), aurora A, aurora B, total or phosphorylated PLK-1 (Thr-210), to phosphorylated H3 (Ser-10), (**C**) total or phosphorylated Bcl-2 (Ser-70), Bax, and Bim. Actin was served as a loading control.

A previous study indicated that the phosphorylation of JNK influenced mitotic arrest and induced cell apoptosis through the Bcl-2 protein family^9^. Thus, we demonstrated that MPT0G066 induced phosphorylation of JNK in a concentration-dependent manner in both ovarian cancer cell lines (Fig. 2A). Furthermore, the regulatory proteins of the G2/M transition were modulated by MPT0G066. After MPT0G066 treatment, the mitotic marker MPM2 and cyclin B were accumulated. MPT0G066 also dephosphorylated cyclin dependent kinase Cdc25 at Ser216, therefore caused a decrease in the levels of inhibitory p-Cdc2 (Tyr15) and increase in the levels of activated p-Cdc2 (Thr161). In addition to the mitotic kinases, PLK1 and Aurora kinases were also activated by MPT0G066 (Fig. 2B). Pro-survival Bcl-2 was phosphorylated and degraded and pro-apoptotic Bax and Bim were accumulated (Fig. 2C). Furthermore, MPT0G066 directly bound to tubulin and depolymerized tubulin structure (Suppplementary Fig. S2). Taken together, MPT0G066 activated JNK phosphorylation, disputed regulatory mitotic proteins, and modulated the Bcl-2 protein family.

### JNK activation is associated with MPT0G066-mediated phosphorylation of Bcl-2, and the apoptosis pathway but not mitotic effects

**Figure 3:**
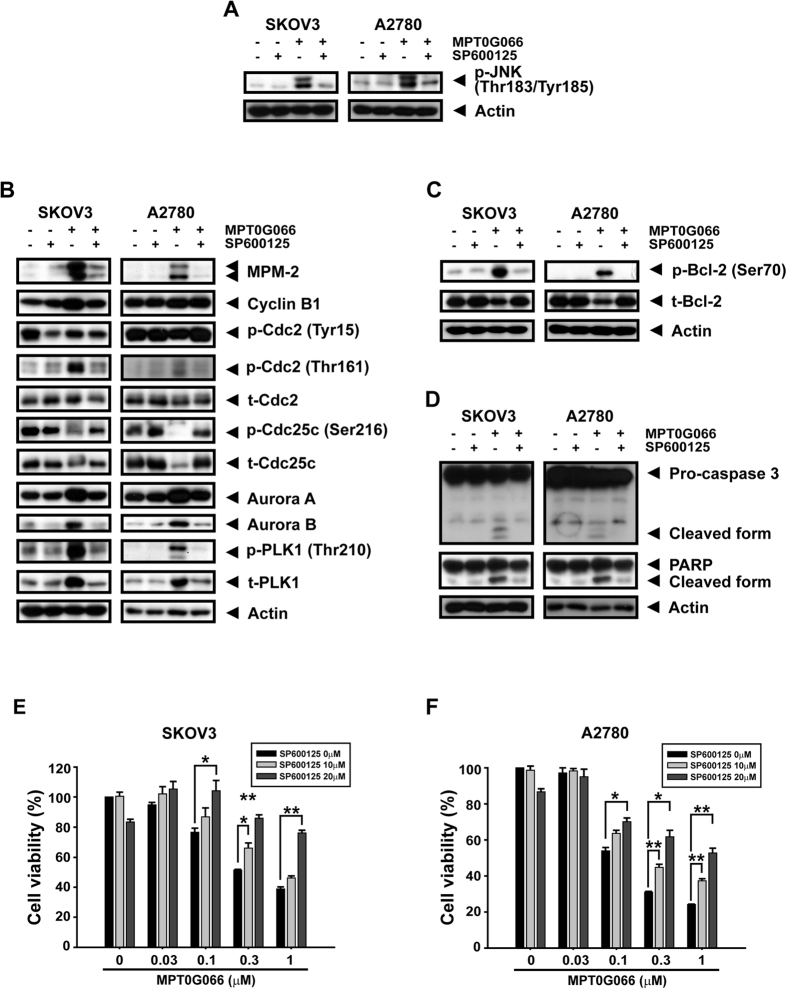
A JNK inhibitor, SP600125, attenuates MPT0G066-mediated JNK activity, regulatory proteins of G2/M and apoptotic pathway. SKOV3 or A2780 cells were treated with 1 μM MPT0G066 with or without 20 μM SP600125 for (**A**–**C**) 24 h or (**D**) 48 h. The whole cell lysates of SKOV3 or A2780 were subject to western blot analysis with (**A**) phosphorylated JNK (Thr-183 and Tyr-185), (**B**) MPM-2, cyclin B1, total or phosphorylated cdc2 (Tyr-15 and Thr-161), total or phosphorylated cdc25c (Ser-216), aurora A, aurora B, total or phosphorylated PLK-1 (Thr-210), (**C**) total or phosphorylated Bcl-2 (Ser-70), (**D**) caspase-3 and PARP. Actin was served as a loading control. (**E**) SKOV3 or (**F**) A2780 cells were incubated with various concentrations of MPT0G066 (0.03–1 μM) with or without 20 μM SP600125 for 48 h. Cell viability was measured by MTT assay.

First, we used a JNK inhibitor, SP600125, to investigate the role of JNK in the antitumor effect of MPT0G066. Fig. 3A showed that SP600125 completely abolished MPT0G066-induced JNK activation. Furthermore, blockage of phosphorylated JNK attenuated the effects of G2/M regulatory proteins, including mitotic markers, cell cycle regulators, and mitotic kinases (Fig. 3B). Bcl-2, a downstream protein of activated JNK, decreased its phosphorylation and degradation (Fig. 3C), and the MPT0G066-triggered apoptosis pathway was reversed by SP600125 (Fig. 3D). In addition, the inhibition of JNK by SP600125 prevented both ovarian cancer cell lines from MPT0G066-decreased cell viability (Fig. 3E,F). However, to our surprise, JNK knockdown using JNK 1/2 siRNA did not reverse the MPT0G066-mediated mitotic proteins expression (Supplementary Fig. S3B); however, it slightly reversed p-Bcl-2 (Ser70), which related to apoptotic effect of MPT0G066 (Supplementary Fig. S3C). Also, JNK knockdown partially reversed the MPT0G066-inhibited cell viability in both cell lines (Supplementary Fig. S3D,E), which consistent with the effect of JNK inhibitor SP600125 treatment and indicated the important role of JNK in MPT0G066-induced cell apoptosis and survival inhibition. Based on these results, we demonstrated that MPT0G066-activated JNK triggered the Bcl-2-related apoptosis pathway but not the mitotic effects.

### MPT0G066 inhibits cell viability in multi-drug resistant ovarian cancer cells

**Figure 4:**
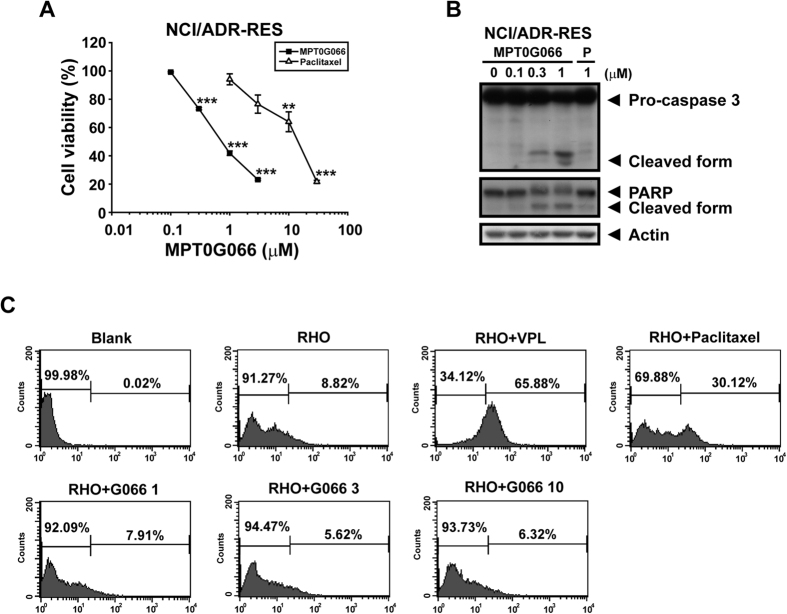
MPT0G066 inhibits cell viability in drug resistant ovarian cancer cell line. (**A**) NCI/ADR-RES cells were incubated with various concentrations of MPT0G066 (0.1–3 μM) or paclitaxel (1–30 μM) for 48 h. Cell viability was analyzed by MTT assay. (**B**) NCI/ADR-RES cells were treated with various concentrations of MPT0G066 (0.1–1 μM) or paclitaxel (1 μM) for 48 h. The whole cell lysates were subject to western blot analysis with caspase-3, PARP and actin. (**C**) NCI/ADR-RES cells were treated with or without indicated agent and co-treated with 1 μM rhodamine 123 (RHO). After 1 h incubation at 37 °C, cells were collected and analyzed by flow cytometry. BLK, blank; VPL, verapamil (50 μM); Paclitaxel (30 μM); G066, MPT0G066 (1, 3, 10 μM).

Drug resistance is an intractable problem in cancer treatment^10^. We used a multi-drug resistant ovarian cancer cell line, NCI/ADR-RES, to investigate whether MPT0G066 could overcome the drug resistance. As shown in Fig. 4A, MPT0G066 displayed the inhibition of cell viability with an IC_50_ value of 0.83 μM for NCI/ADR-RES cells in the MTT assay, but paclitaxel only presented a moderate effect with an IC_50_ value of 11.02 μM. Also, MPT0G066 induced a caspase-dependent apoptosis pathway in NCI/ADR-RES cells (Fig. 4B).

Furthermore, we used rhodamine 123, a dye of p-glycoprotein (p-gp) substrate, to compete with MPT0G066 to determine whether MPT0G066 would be pumped out by p-gp. As shown in Fig. 4C, co-treatment of rhodamine 123 with verapamil, a p-gp inhibitor, revealed the accumulation of rhodamine 123 in NCI/ADR-RES cells. However, paclitaxel, a well-known p-gp substrate, competed the binding to p-gp with rhodamine 123 and therefore increased the accumulation of rhodamine 123. In contrast, even 10 μM of MPT0G066 did not cause the retention of rhodamine 123. As a result, MPT0G066 effectively induced apoptosis in the multi-drug resistant cell line NCI/ADR-RES without interfering with p-gp.

### Combination of MPT0G066 and cisplatin shows synergetic antitumor activity in ovarian cancer cells *in vitro*

**Figure 5:**
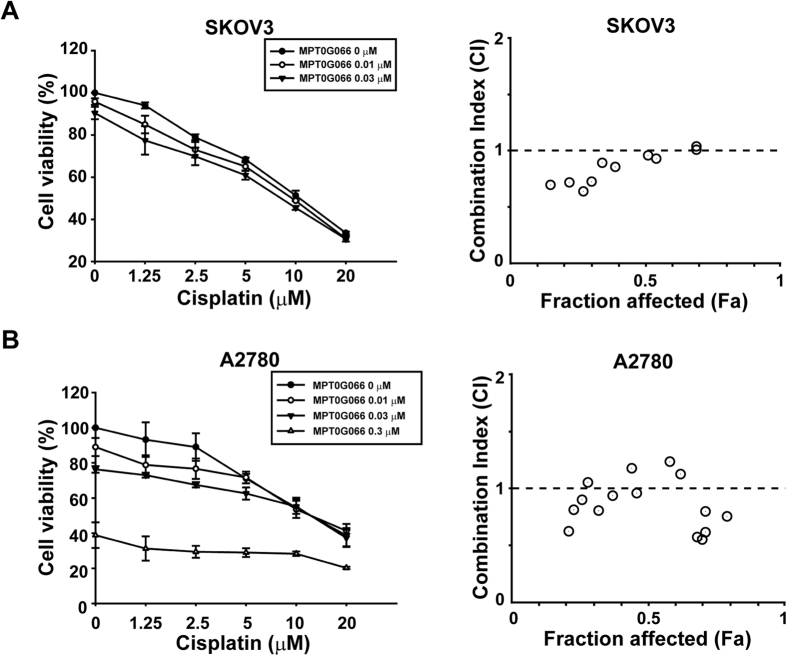
MPT0G066 potentiates antineoplastic effects of cisplatin in ovarian cancer *in vitro*. (**A**) SKOV3 and (**B**) A2780 cells were incubated with MPT0G066 (0.01–0.3 μM) and cisplatin alone or increasing concentration (1.25–20 μM) for 48 h. *Left panel:* cell viability was determined by MTT assay. *Right panel:* CI values for combination of MPT0G066 and cisplatin. The resulting combination index (CI) theorem of Chou-Talalay offers quantitative definition for additive effect (CI = 1), synergetic effect (CT > 1), and antagonism effect (CI < 1) in drug combinations. Fa represented fraction of cells affected.

For exploration of the extended clinical usage of MPT0G066, we examined the antitumor activity of the combination of MPT0G066 and cisplatin. Cisplatin is the first-line therapy against ovarian cancer, but causes nephrotoxicity and neurotoxicity^1^. As shown in Fig. 5 and Supplementary Table S1, MPT0G066 potentiated the antitumor activity of low-dose cisplatin and combination index (CI) values represented the synergistic effects in both ovarian cancer cell lines. This result demonstrated that MPT0G066 possessed effective antitumor activity for single or combination treatment with cisplatin and may decrease cisplatin-induced toxicity.

### MPT0G066 shows antitumor activity alone or with cisplatin in human ovarian cancer xenograft models

**Figure 6:**
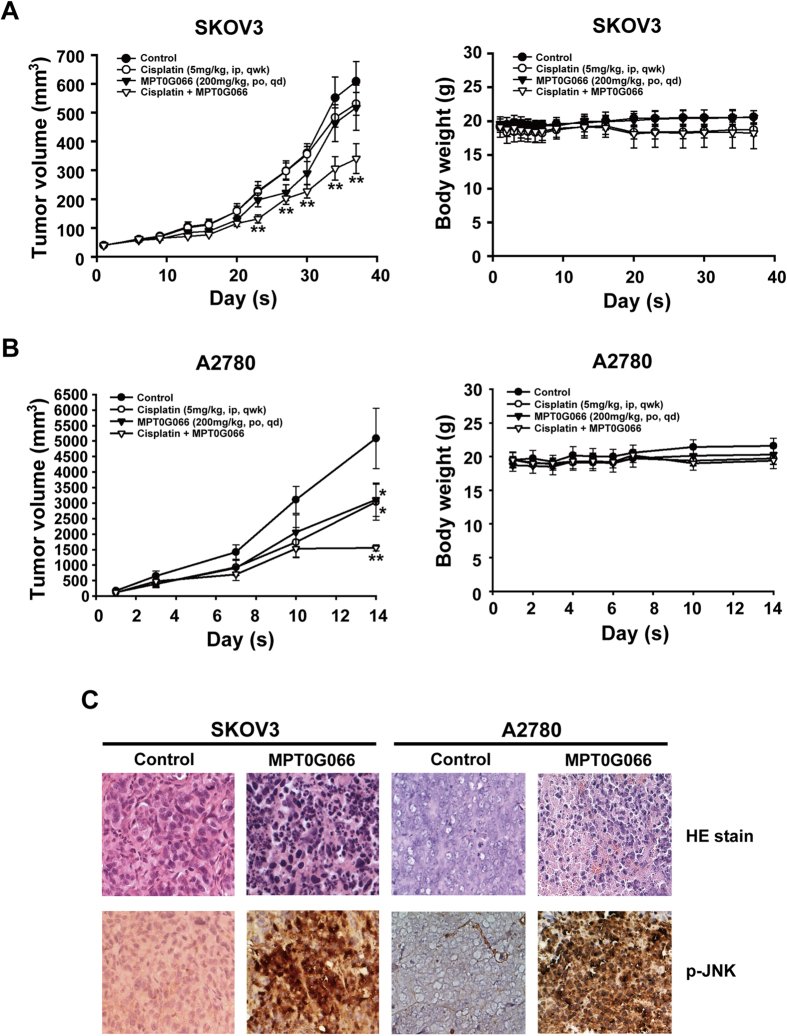
MPT0G066 suppresses human ovarian carcinoma growth and potentiates antineoplastic effects of cisplatin in ovarian cancer *in vivo*. SKOV3 and A2780 xenograft nude mice were orally (po) treated with MPT0G066 (200 mg/kg) daily or intravenously (iv) administered cisplatin (5 mg/kg) once a week or combination or vehicle after tumor cell implantation. Tumor volume and Body weight of SKOV3 and A2780 xenografts were shown in (**A**,**B**). (**C**) Formalin-fixed paraffin-embedded sections from SKOV3 and A2780 were stained with H&E stain and phosphorylated JNK (Thr-183 and Tyr-185) antibody. The stained sections were used 200X magnification to observe via microscope.

Given our *in vitro* results, we further investigated the antitumor activity of MPT0G066 *in vivo* in xenograft animal models. The results demonstrated that co-treatment of MPT0G066 with cisplatin significantly reduced the tumor volume without affecting body weight in both human ovarian cancer xenograft models (Fig. 6A,B). Moreover, we used immunohistochemistry analysis to examine the antitumor activity of MPT0G066 *in vivo*. H&E staining showed that MPT0G066 directly increased nuclear condensation and apoptotic cells in both ovarian cancer tissues. An increase in phosphorylated JNK levels (brown color) was observed in MPT0G066-treated groups (Fig. 6C). Therefore, MPT0G066 could decrease ovarian tumor growth *in vivo*.

## Discussion

Ovarian cancer is a heterogeneous disease, exhibiting diverse genetic alterations, and tumor behaviors^2^,^12^. Aneuploidy, which is the presence of an abnormal number of chromosomes in a cell, is associated with tumor progression and survival in ovarian cancer^13^,^14^. In addition, p53 mutations and abnormalities are associated with an increase in the number of aneuploid malignant cells^15^. In this study, MPT0G066 displayed a cytotoxic effect on two different ovarian cancer cells *in vitro* and *in vivo* (Figs 1 and 6). A2780 with wild type p53 is an untreated ovarian cancer cell line and sensitive to chemotherapy^16^; while SKOV3 is a near-tetraploid ovarian cancer cell line with mutant p53, and resistant to platinum agents^17^. Accumulation of polyploidy cells (MP phase) may cause aberrant chromosome segregation without functional p53 and generate genomic instability, resulting in mitotic-linked cell death, also called mitosis catastrophe^9^,^18^. Thus, the treatment of MPT0G066 on SKOV3 cells led to a high proportion of cells in the MP phase; however, this effect was not seen in A2780 cells (Fig. 1D,E). Taken together, these results suggested that MPT0G066 has antitumor capability in both platinum-sensitive and resistant ovarian cancer cells in a p53-independent manner.

Mitosis is a complex and delicate process. During M-phase entry, the increase in the levels of MPM2 and reduction in the levels of inhibitory cdc2 (Tyr-15) promotes the binding of activated cdc2 (Thr-161) to cyclin B1, resulting in cell cycle progression. In order to link microtubules and chromosomes correctly, Aurora kinases and PLK1 regulate the formation of the mitotic spindle, the arrangement of chromosomes and separation of chromosomes. However, incorrect linkage between microtubules and chromosomes can activate the mitotic spindle checkpoint and prevent mitosis process^8^,^9^,^18^. MPT0G066, like vincristine, induced microtubule disassembly and disrupted microtubule dynamics (Supplementary Fig. S2), followed by the prolonged activation of the mitotic check-point, including Aurora kinases and PLK1 (Fig. 2B), and the arrest of the cell cycle in the MP or G2/M phase (Fig. 1D,E). However, the dose-response effect of MPT0G066 on mitotic proteins is slightly different in two cell lines. The possible explanation is that the therapeutic window of MPT0G066 in A2780 is narrower compared with that in SKOV3. Therefore, the dose-response effect of MPT0G066 on mitotic proteins and also apoptotic proteins would be sharper in A2780 compared with that in SKOV3. However, despite the fact that MPT0G066 induced different dose-response in two cells, MPT0G066 is a microtubule-interfering agent and leads to cell cycle arrest in the G2/M phase.

The intrinsic apoptotic pathway is an apoptotic pathway involved in the loss of mitochondrial membrane potential via dysregulation of the Bcl-2 protein family^3^. Previous studies indicate that microtubule-interfering agents, including paclitaxel and vinblastine^5^,^6^,^7^, also cause degradation of Bcl-2 through the increase in the levels of phosphorylated Bcl-2, which triggers the activation of downstream apoptotic cascades^9^. During mitotic checkpoint activation, MPT0G066 caused a decrease in Bcl-2 levels, but increased the levels of pro-apoptotic proteins, Bax and Bim (Fig. 2). The apoptotic pathway was activated by cleavage of caspase-3 and PARP (Fig. 1F). Moreover, JNK activation may promote dissociation of Bim from the disrupted microtubules into the cytoplasm and degradation of Bcl-2, resulting in apoptosis^9^,^19^. Inhibition of JNK activation by SP600125 and JNK siRNA reversed MPT0G066-mediated dysregulation of the Bcl-2 protein family and apoptosis (Fig. 3 and Supplementary Fig. S3). Taken together, MPT0G066-induced apoptosis and the dysregulation of Bcl-2 protein family function is mediated by JNK activation.

JNK signaling plays a physiological role in cell cycle progression. Based on our results, MPT0G066-regulated mitotic proteins could not be reversed by JNK knockdown and is not consistent with the reverse effect of SP600125, a JNK inhibitor, which may due to the specific function of SP600125. Several studies demonstrated that the inhibition of basal JNK activity by SP600125 caused abnormal cell replication and cell death^20^,^21^. However, another study revealed that SP600125 directly inhibited Aurora-A, PKL1 and cdk1 activity to promote cell endoreplication without mitosis in a JNK-independent manner^22^. Thus, SP600125 may regulate the cell cycle at the G2/M phase in both JNK-dependent and -independent manners. According to our unpublished data, SP600125 alone would increase the cell population of G2/M phase in both cell lines without affecting mitotic regulatory proteins (Fig. 3B), which might indicated that SP600125 reverse MPT0G066-regulated mitotic proteins by promoting cell endoreplication in a JNK-independent manner. On the contrary, microtubule-interfering agent-mediated apoptosis could be attenuated by SP600125 and JNK knockdown using siRNA for JNK signaling inhibition^7^. In addition, inhibition of JNK by dominant-negative JNK (DN-JNK) also abolished paclitaxel-induced acute cell death in ovarian cancer^23^. In this study, MPT0G066, a microtubule-interfering agent (Supplementary Fig. S2), activated JNK signaling and cell death, which was attenuated by SP600125 and JNK knockdown (Fig. 3). Therefore, our data indicated that MPT0G066 indeed induce JNK-mediated apoptosis but regulate mitotic process in a JNK-independent manner. Accordingly, JNK is important for the induction of apoptosis following the stress of microtubule disruption under MPT0G066 treatment.

Drug resistance is a notable medical issue in cancer treatment. The mechanisms of drug resistance in chemotherapy can involve p-gp expression, metabolic enzymes, and survival signaling from the tumor microenvironment. Overexpression of p-gp causes efflux of taxane drugs, thereby reducing the concentration of free drug in cancer cells. The increase in glutathione synthesis can deactivate platinum drugs, resulting in drug resistance^10^,^16^. Our results demonstrate that the antitumor activity of MPT0G066 was 13.28-fold more potent than the antitumor activity of paclitaxel, and MPT0G066 effectively promotes the apoptotic pathway without the influence of p-gp in multi-drug resistant ovarian cancer cell lines NCI/ADR-RES (Fig. 4). In addition, the combination of MPT0G066 and cisplatin revealed a potentiated cytotoxic effect in both platinum-resistant and platinum-sensitive ovarian cancer cells *in vitr*o and *in vivo* (Figs 5 and 6A,B). This combination strategy may decrease the toxicity of cisplatin and increase the therapeutic safety. Further, MPT0G066 showed weak inhibition of cell viability with a 13- to 14- folds difference in normal cells HUVEC (Supplementary Fig. S1). As a result, MPT0G066 may be a novel antineoplastic compound and have a more selective effect against chemo-sensitive or chemoresistant ovarian cancer cells.

In summary, we demonstrated that MPT0G066 induced JNK activation, resulting in the JNK-independent cell cycle arrest in MP or G2/M phases, and altering Bcl-2 family proteins, which ultimately caused apoptosis through the intrinsic apoptotic pathways. In contrast to traditional chemotherapy, MPT0G066 retained its potent cytotoxic effect in multi-drug resistant cell line NCI/ADR-RES without being a p-gp substrate. Additionally, the combination of MPT0G066 and cisplatin showed synergistic antitumor activity *in vitro* and *in vivo*. Taken together, we suggest that MPT0G066 may be an additional strategy against ovarian cancer.

## Materials and Methods

### Reagents

MPT0G066 (Fig. 1A) was obtained from Professor Jing-Ping Liou (College of Pharmacy, Taipei Medical University, Taipei, Taiwan). RPMI-1640 medium, DMEM medium, fetal bovine serum (FBS), penicillin, streptomycin and all other culture reagents were obtained from GIBCO/BRL Life Technologies (Grand Island, NY, USA). Sulforhodamine B (SRB), 3-(4,5-dimethylthiazol-2-yl)-2,5-diphenyltetrazolium bromide (MTT), propidium iodide (PI), anti-β-tubulin, FITC-conjugated anti-mouse IgG and SP600125 were ordered from Sigma Chemical (St Louis, MO, USA). Antibodies against capase-7, and phosphorylated PLK (Thr-210) were purchased from BD Biosciences (San Jose, CA, USA). Antibody against caspase-3 was purchased from Imgenex (San Diego, CA, USA). Antibodies specific for phosphorylated cdc2 (Thr-161 and Tyr-15), Aurora A, Aurora B, phosphorylated cdc25C (Ser-216), PLK1, JNK, phosphorylated Bcl-2 (Ser-70) and phosphorylated H3 (Ser-10) antibodies were obtained from Cell Signaling Technology (Beverly, MA, USA). Actin antibody was purchased from Chemicon (Billerica, MA, USA). Antibody to PARP, cyclin B1, cdc2, cdc25C, Bcl-2, Mcl-1, Bax, Bim, PUMA, HRP-conjugated anti-mouse and anti-rabbit IgGs were ordered from Santa Cruz Biotechnology (Santa Cruz, CA, USA). Phosphorylated MPM2 (Ser/Thr) antibody was bought from Upstate Biotechnology Inc. (Temecula, CA, USA). Phosphorylated JNK (Thr-183 and Tyr-185) was obtained from CalBiochem (Darmstadt, Germany).

### Cell culture

Human ovarian cancer cell lines SKOV3 was purchased from American Type Culture Collection (ATCC; Manassas, VA, USA); A2780 was bought from Sigma Chemical (St Louis, MO, USA); NCI/ADR-RES was obtained from the Developmental Therapeutics Program Human Tumor Cell Line Screen (National Cancer Institute). SKOV3 cells were maintained in DMEM mediums with 10% FBS (v/v); A2780 and NCI/ADR-RES cells were cultured in RPMI-1640 with 10% FBS (v/v). HUVECs were maintained in M199 contained with 20% FBS (v/v). Both mediums were supplemented with 100 U/mL penicillin, 100 μg/mL streptomycin, and 2.5 μg/mL amphotericin B. Cells were maintained in a humidified incubator at 37 °C in 5% CO_2_/95% air.

### Sulforhodamine B (SRB) assay

Counted 5000 cells and then seeded into 96-wells for overnight. Basal cells were fixed by 10% trichloroacetic acid (TCA), called T_0_. Other cells were treated with indicated concentrations of MPT0G066 for 48 h in 5% culture medium. After fixed with 10% TCA, cells were stained with 0.4% SRB and then washed by 1% acetic acid three times. Cells were lysed by 10 mM Trizma base and then the absorbance was detected by an ELISA reader at 515 nm wavelength. The 50% growth inhibition (GI_50_) was calculated by 100 − [(T_x_ − T_0_)/(C − T_0_)] × 100.

### Cell viability assay

Cells were seeded into 96-wells for overnight and then treated with indicated concentrations of MPT0G066, cisplatin or both drugs for different time intervals. Washed out and incubated with medium contained 0.5 mg/mL MTT for 1 h. Cells were lysed by DMSO and then the absorbance was detected by an ELISA reader at 550 nm wavelength. The 50% of inhibitory concentration (IC_50_) was calculated by the ratio (%) of absorbance between control and treatment group. Combination index (CI) and fractional effect (Fa) were measured by Compusyn software (ComboSyn, Inc., Paramus, NJ. USA).

### FACScan Flow cytometry

Cells were seeded into 6-wells for overnight. After adherence, cells were treated with indicated concentration of MPT0G066 for different time intervals and then collected by trypsinization, fixed with 75% (v/v) ethanol at −20 °C overnight. After centrifugation, cells were incubated in phosphate-citric acid buffer (NaHPO_4_ 0.2 M, citric acid 0.1 M (pH 7.8)) for 20 minutes at room temperature. Then, cells were centrifuged and resuspended with 0.5 mL PI solution (Triton X-100 0.1%, RNase 100 μg/mL and PI 80 μg/mL). DNA content was analyzed with the FACScan and CellQuest software (Becton Dickinson).

For p-glycoprotein (p-gp) activity assay, multi-drug resistant NCI/ADR cells were co-treated with indicated agents and 10 μM rhodamine 123 for 60 min. Then, cells were washed by PBS and collected by trypsinization. The results were detected with the FACScan and CellQuest software (Becton Dickinson).

### Western blot analysis

Whole cell lysate was extracted by a lysis buffer (Tris 20 mM (pH 7.5), NaCl 150 mM, EDTA 1mM, EGTA 1mM, Triton X-100 1% and sodium pyrophosphate 2.5 mM) for 30 min at 4 °C and then centrifuged at 13,000 rpm at 4 °C for 30 min. Protein was quantified by BCA Protein Assay Kit (Thermo scientific, Rockford, IL, USA). Equal protein amounts of protein was resolved by 10% SDS-polyacrylamide gel, and then transferred onto a nitrocellulose membrane after electrophoresis. The membranes were incubated with specific primary antibodies overnight at 4 °C and then applied to appropriate secondary antibodies for 1 h. Detection of signal was performed with an enhanced chemiluminescence detection kit (Amersham, England).

### *In vitro* tubulin polymerization assay

CytoDYNAMIX Screen 03 Kit (Cytoskeleton Inc.) was used to perform cell-free tubulin polymerization assay. General tubulin buffer, GTP stock (100 mM), tubulin protein (10 mg/ml) and tubulin polymerization (TP) buffer were all prepared on ice and followed by manufacture’s instruction. Next, indicated concentrations of drugs (2 μl) were added into each eppendorf included 85 μl TP buffer. Paclitaxel and vincristine were used as positive controls. Finally, 30 μl tubulin protein was added into each eppendorf and transferred to the prewarmed 96-well plate. The absorbance was measured by a spectrophotometer and recorded every 1 min for 30 min at 340 nm and 37 °C.

### Immunofluorescence

Cells were seeded on chamber slide and treated with indicated condition. Cells were washed with PBS, fixed with −20 °C methanol at 4 °C for 10 min and then blocked with 2% BSA in PBS at 37 °C for 1 h. After PBS washing, cells were incubated with indicated primary antibody at 4 °C overnight. After PBS washing, secondary antibody FITC-conjugated DAPI containing IgG were added and incubated for 45 min at 4 °C in dark. Slides were imaged using a Leica TCS SP2 confocal spectral microscope (Buffalo, NY, USA).

### Transient transfection

OnTARGET PLUS SMART pool JNK1 and JNK2 siRNA was purchased from Dharmacon (Lafayette, CO, USA). SKOV3 and A2780 cells were transfected with Lipofectamine RNAiMAX transfection reagent (Invitrogen) according to the manufacturer’s instruction. Transfected cells were grown for 24 h, treated with MPT0G066 for another 24 h and then harvested for western blot analysis or for another 48 h and subjected to MTT assay for cell viability determination.

### *In vivo* xenograft model

The human ovarian cancer cell SKOV3 and A2780 used for implantation were injected subcutaneously (s.c.) with 10^8^ cells into 4-week-old female balb/c nude mice. After tumor volume about 50–100 mm^3^, mice were divided into 4 groups: vehicle control, 200 mg/kg MPT0G066 orally daily (dissolved in 0.5% carboxymethyl cellulose, 0.1% tween 80, 5% dextrose), 5 mg/kg cisplatin intravenously once a week and combination of MPT0G066 and cisplatin. Tumor volumes were monitored daily at first week and then twice weekly until tumor volumes of control group approached maximum. Tumor volume was calculated from (W^2^ × L)/2. W, width and L, length. Animal experiments were performed in accordance with relevant guidelines and regulations followed ethical standards, and protocols has been reviewed and approved by Animal Use and Management Committee of Taipei Medical University (LAC-2013–0139).

### Immunohistochemistry (IHC)

Formalin-fixed paraffin-embeded tumor sections were deparaffinized by xylene and then rehydration by different alcohol concentration (form 100%, 95%, 75%, 50% and ddH_2_O). Then, the slides were immersed in 3% H_2_O_2_ to inactivate endogenous peroxidase. For antigen retrival, slides were incubated in buffer with pH 9 (Tris 0.605g, EDTA disodium 0.263g, 0.05% tween 20 in 500mL ddH_2_O) at 80 °C overnight. After PBS washing, the slides were blotting by 4% fatty-free milk for 30 mins at room temperature and then incubated with primary antibody at 4 °C overnight. The secondary antibody, HRP Polymer Conjugate Reagent (SuperPicture Polymer Detection kit), were added for 10 min, and then Mayer’s Hematoxylin solution was used for counterstaining. For hematoxylin and eosin stain (H&E stain), the tumor sections were incubated in hematoxylin solution for 15 min and then counterstained with eosin for 5 min. All of slides were observed by 200x magnification through microscopy.

### Statistics

Every experiment was performed at least three times. All data represent the mean ± S.E.M. from three independent experiments. Statistical analysis was using Student’s *t*-Test. One asterisk indicates *P* < 0.05, two asterisks indicate *P* < 0.01, and three asterisks indicate *P* < 0.001.

## Additional Information

**How to cite this article**: Huang, H.-L. *et al*. MPT0G066, a novel anti-mitotic drug, induces JNK-independent mitotic arrest, JNK-mediated apoptosis, and potentiates antineoplastic effect of cisplatin in ovarian cancer. *Sci. Rep.* **6**, 31664; doi: 10.1038/srep31664 (2016).

## Supplementary Material

Supplementary Information

## References

[b1] Jelovac D. & Armstrong D. K. Recent progress in the diagnosis and treatment of ovarian cancer. CA Cancer J. Clin. 61, 183–203 (2011). 21521830 10.3322/caac.20113 PMC3576854

[b2] Karst A. M. & Drapkin R. Ovarian cancer pathogenesis: a model in evolution. J. Oncol. 2010, 932371 (2010). 19746182 10.1155/2010/932371 PMC2739011

[b3] Dhanasekaran D. N. & Reddy E. P. JNK signaling in apoptosis. Oncogene 27, 6245–6251 (2008). 18931691 10.1038/onc.2008.301 PMC3063296

[b4] Lopez-Bergami P. The role of mitogen- and stress-activated protein kinase pathways in melanoma.Pigment Cell Melanoma Res 24, 902–921 (2011). 21914141 10.1111/j.1755-148X.2011.00908.x

[b5] Wang T. H. . Microtubule-interfering agents activate c-Jun N-terminal kinase/stress-activated protein kinase through both Ras and apoptosis signal-regulating kinase pathways.J. Biol. Chem. 273, 4928–4936 (1998). 9478937 10.1074/jbc.273.9.4928

[b6] Fan M. . Vinblastine-induced phosphorylation of Bcl-2 and Bcl-XL is mediated by JNK and occurs in parallel with inactivation of the Raf-1/MEK/ERK cascade. J. Biol. Chem. 275, 29980–29985 (2000). 10913135 10.1074/jbc.M003776200

[b7] Kolomeichuk S. N., Terrano D. T., Lyle C. S., Sabapathy K. & Chambers T. C. Distinct signaling pathways of microtubule inhibitors--vinblastine and Taxol induce JNK-dependent cell death but through AP-1-dependent and AP-1-independent mechanisms, respectively. FEBS J. 275, 1889–1899 (2008). 18341588 10.1111/j.1742-4658.2008.06349.x

[b8] Jackson J. R., Patrick D. R., Dar M. M. & Huang P. S. Targeted anti-mitotic therapies: can we improve on tubulin agents? Nat. Rev. Cancer 7, 107–117 (2007). 17251917 10.1038/nrc2049

[b9] Mollinedo F. & Gajate C. Microtubules, microtubule-interfering agents and apoptosis. Apoptosis 8, 413–450 (2003). 12975575 10.1023/a:1025513106330

[b10] Agarwal R. & Kaye S. B. Ovarian cancer: strategies for overcoming resistance to chemotherapy. Nat. Rev. Cancer 3, 502–516 (2003). 12835670 10.1038/nrc1123

[b11] Lai M.-J. . N-Sulfonyl-aminobiaryls as Antitubulin Agents and Inhibitors of Signal Transducers and Activators of Transcription 3 (STAT3) Signaling. J. Med. Chem. 58, 6549–6558 (2015). 26241032 10.1021/acs.jmedchem.5b00659

[b12] Wang V. . Ovarian cancer is a heterogeneous disease. Cancer Genet. Cytogenet. 161, 170–173 (2005). 16102589 10.1016/j.cancergencyto.2004.12.014

[b13] Friedlander M. L. . Influence of cellular DNA content on survival in advanced ovarian cancer. Cancer Res. 44, 397–400 (1984). 6690054

[b14] Rodenburg C. J., Cornelisse C. J., Heintz P. A., Hermans J. & Fleuren G. J. Tumor ploidy as a major prognostic factor in advanced ovarian cancer. Cancer 59, 317–323 (1987). 3802018 10.1002/1097-0142(19870115)59:2<317::aid-cncr2820590225>3.0.co;2-4

[b15] Kihana T. . High incidence of p53 gene mutation in human ovarian cancer and its association with nuclear accumulation of p53 protein and tumor DNA aneuploidy. Jpn. J. Cancer Res. 83, 978–984 (1992). 1429209 10.1111/j.1349-7006.1992.tb02010.x PMC5918982

[b16] Godwin A. K. . High resistance to cisplatin in human ovarian cancer cell lines is associated with marked increase of glutathione synthesis. Proc. Natl. Acad. Sci. USA 89, 3070–3074 (1992). 1348364 10.1073/pnas.89.7.3070 PMC48805

[b17] Hills C. A. . Biological properties of ten human ovarian carcinoma cell lines: calibration *in vitro* against four platinum complexes. Br. J. Cancer 59, 527–534 (1989). 2653399 10.1038/bjc.1989.108 PMC2247140

[b18] Castedo M. . Cell death by mitotic catastrophe: a molecular definition. Oncogene 23, 2825–2837 (2004). 15077146 10.1038/sj.onc.1207528

[b19] Lei K. & Davis R. J. JNK phosphorylation of Bim-related members of the Bcl2 family induces Bax-dependent apoptosis. Proc. Natl. Acad. Sci. USA 100, 2432–2437 (2003). 12591950 10.1073/pnas.0438011100 PMC151358

[b20] Miyamoto-Yamasaki Y., Yamasaki M., Tachibana H. & Yamada K. Induction of endoreduplication by a JNK inhibitor SP600125 in human lung carcinoma A 549 cells. Cell Biol. Int. 31, 1501–1506 (2007). 17904874 10.1016/j.cellbi.2007.07.002

[b21] Moon D. O. . JNK inhibitor SP600125 promotes the formation of polymerized tubulin, leading to G2/M phase arrest, endoreduplication, and delayed apoptosis. Exp. Mol. Med. 41, 665–677 (2009). 19478553 10.3858/emm.2009.41.9.073 PMC2753660

[b22] Kim J. A., Lee J., Margolis R. L. & Fotedar R. SP600125 suppresses Cdk1 and induces endoreplication directly from G2 phase, independent of JNK inhibition. Oncogene 29, 1702–1716 (2010). 20062077 10.1038/onc.2009.464 PMC3145494

[b23] Wang T. H. . Microtubule dysfunction induced by paclitaxel initiates apoptosis through both c-Jun N-terminal kinase (JNK)-dependent and -independent pathways in ovarian cancer cells. J. Biol. Chem. 274, 8208–8216 (1999). 10075725 10.1074/jbc.274.12.8208

