# Supplementary material for: MPT0G066, a novel anti-mitotic drug, induces JNK-independent mitotic arrest, JNK-mediated apoptosis, and potentiates antineoplastic effect of cisplatin in ovarian cancer
Source: Sci Rep. 2016 Aug 16;6:31664. doi: 10.1038/srep31664 (PMC4985652; doi:10.1038/srep31664)
Supplement: Supplementary Information [file srep31664-s1.pdf]

Supplementary Figures

MPT0G066, a novel anti-mitotic drug, induces JNK-independent mitotic arrest, JNK-mediated apoptosis, and potentiates antineoplastic effect of cisplatin in ovarian cancer

Han-Li Huang[1], Min-Wu Chao[1], Ya-Chi Li[2], Li-Hsun Chang[2], Chun-Han Chen[3], Mei-Chuan Chen[4,5], Chun-Chun Cheng[1], Jing-Ping Liou[6], Che-Ming Teng[2] and Shiow-Lin Pan[1,3,*]

[1] *The Ph.D. Program for Cancer Biology and Drug Discovery, College of Medical Science and Technology, Taipei Medical University, Taipei, Taiwan*

[2]*Pharmacological Institute, College of Medicine, National Taiwan University, Taipei, Taiwan*

[3]*Department of Pharmacology, School of Medicine, College of Medicine, Taipei Medical University, Taipei, Taiwan*

[4]*Ph.D. Program for the Clinical Drug Discovery from Botanical Herbs, College of Pharmacy, Taipei Medical University, Taipei, Taiwan.*

[5]*Graduate Institute of Pharmacognosy, College of Pharmacy, Taipei Medical University, Taipei, Taiwan.*

[6]*School of Pharmacy, College of Pharmacy, Taipei Medical University, Taipei, Taiwan*

***Correspondence:**

Shiow-Lin Pan, Ph.D.
The Ph.D. Program for Cancer Biology and Drug Discovery,
College of Medical Science and Technology, Taipei Medical University,
No. 250 Wuxing Street, Taipei 11031, Taiwan
E-mail: slpan@tmu.edu.tw
Phone: 886-2-27361661 ext 7671

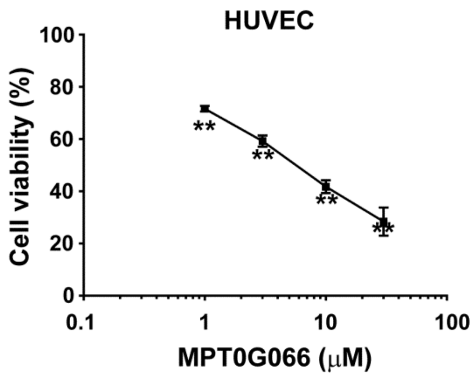

Supplementary Fig. S1. MPT0G066 inhibits cell viability in human umbilical vein endothelial cells (HUVECs). HUVEC were treated with various concentrations of MPT0G066 (1-30 μM) for 48 h. Cell viability was analyzed by MTT assay.

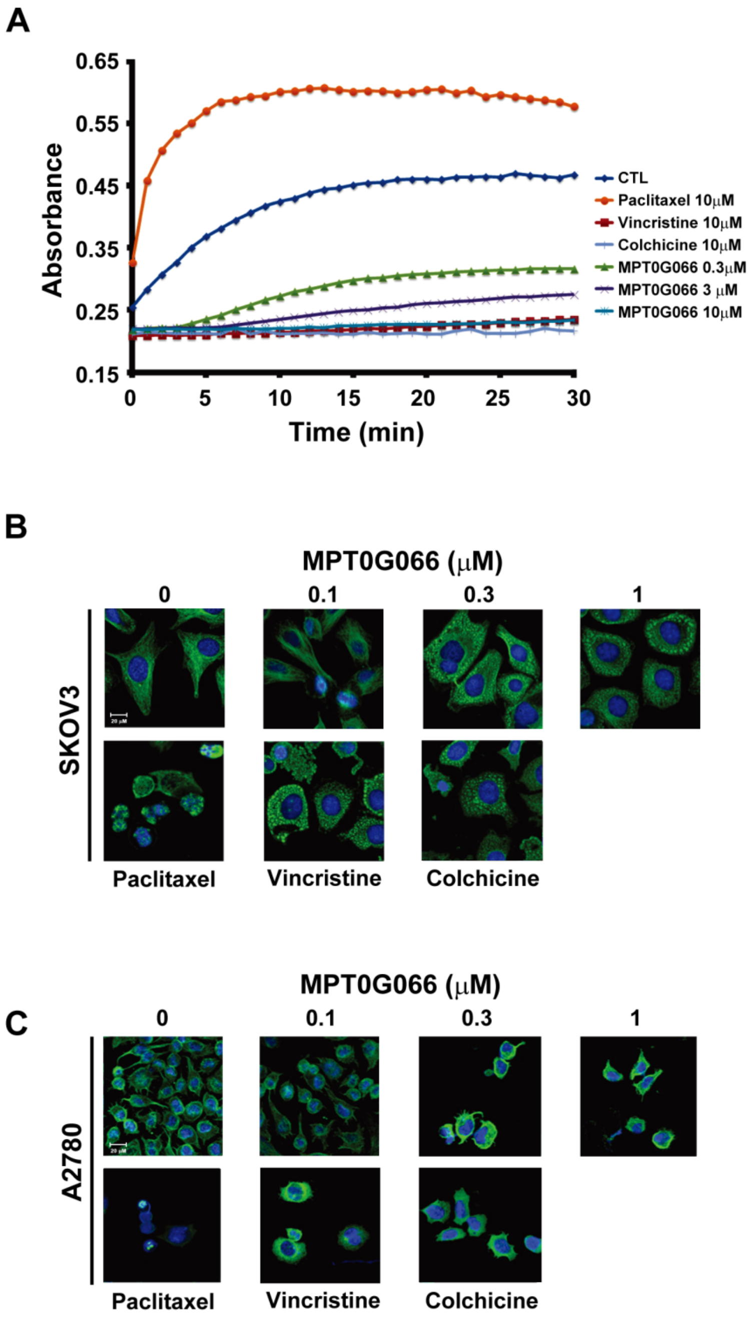

Supplementary Fig. S2. MPT0G066 is a microtubule interfering agent and suppresses tubulin polymerization. (A) Tubulin in reaction buffer was incubated at 37°C with vehicle, the indicated concentrations of MPT0G066 (0.3, 3 and 10 μM), paclitaxel (10 μM), vincristine (10 μM) or colchicine (10 μM). Then, microtubule

assembly was measured by spectrophotometry. (B) SKOV3 and (C) A2780 were incubated with vehicle, the various concentrations of MPT0G066 (0.1-3 μM), paclitaxel (0.03 μM), vincristine (0.03 μM) or colchicine (1 μM) for 18 h. The cellular microtubule network was analyzed by confocal microscopy using monoclonal anti-β-tubulin antibody, FITC-conjugated anti-mouse antibody, and counterstaining with DAPI. Scale bar, 20 μm.

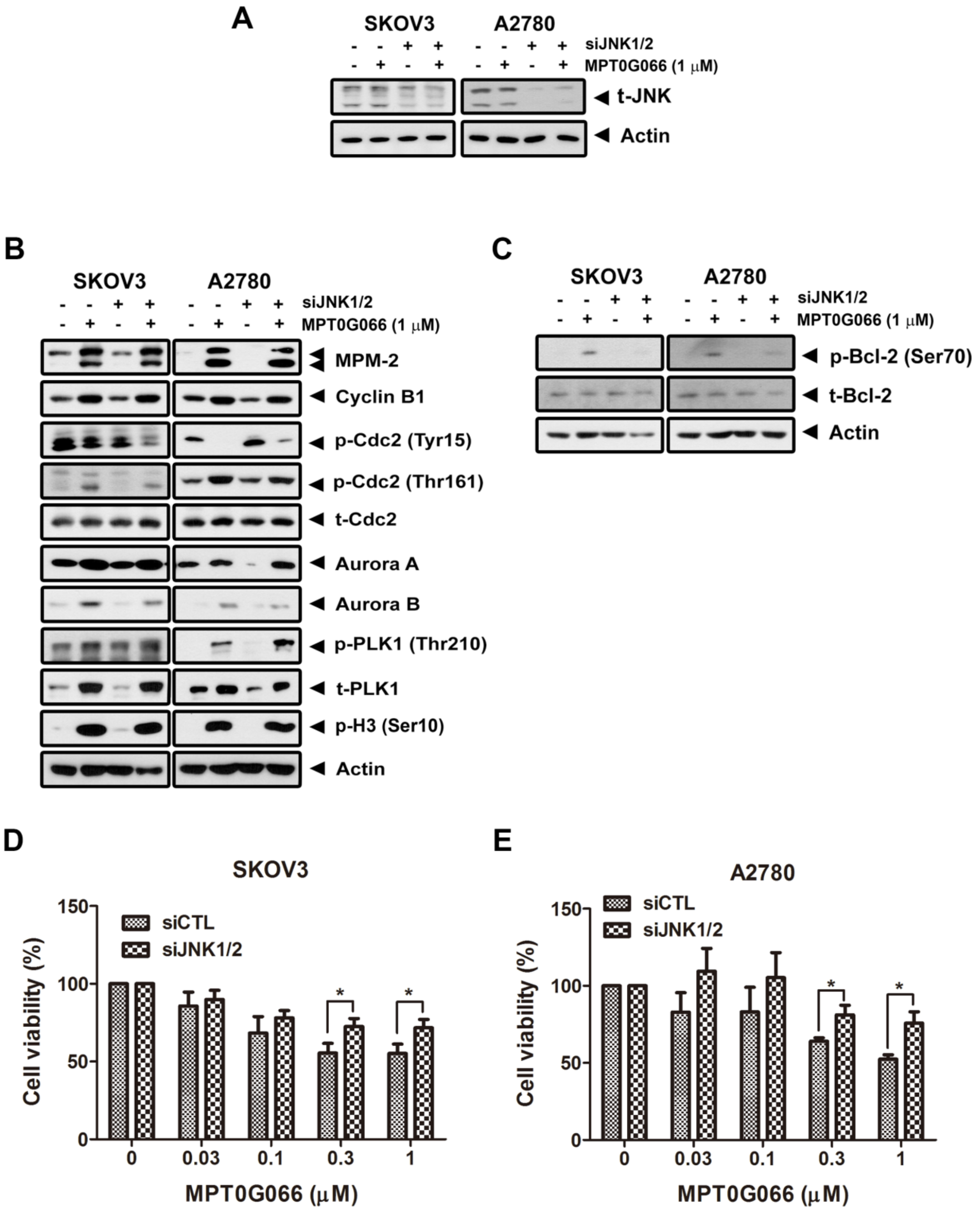

Supplementary Fig. S3. The role of JNK in MPT0G066-regulated mitotic and apoptotic proteins and MPT0G066-inhibted ovarian cancer cell viability. SKOV3 and A2780 were transiently transfected with JNK1/2 siRNA for 24 h followed by additional MPT0G066 treatment for (A, B and C) 24 h and collected for western blot

analysis, or (D and E) 48 h for cell viability evaluation using MTT assay.

Supplementary Table S1. Combination index (CI) values of MPT0G066 in combination with cisplatin in ovarian cancer cells

SKOV3			A2780		
MPT0G066 (μM)	Cisplatin (μM)	CI value	MPT0G066 (μM)	Cisplatin (μM)	CI value
0.01	1.25	0.702	0.01	1.25	0.63
0.01	2.5	0.638	0.01	2.5	0.82
0.01	5.0	0.894	0.01	5.0	1.05
0.01	10.0	0.961	0.01	10.0	0.96
0.01	20.0	1.01	0.01	20.0	1.24
0.03	1.25	0.72	0.03	1.25	0.91
0.03	2.5	0.73	0.03	2.5	0.81
0.03	5.0	0.86	0.03	5.0	0.94
0.03	10.0	0.93	0.03	10.0	1.18
0.03	20.0	1.04	0.03	20.0	1.13
			0.3	1.25	0.58
			0.3	2.5	0.56
			0.3	5.0	0.62
			0.3	10.0	0.80
			0.3	20.0	0.76
